# Demystifying the effect of receptive field size in U-Net models for medical image segmentation

**DOI:** 10.1117/1.JMI.11.5.054004

**Published:** 2024-10-29

**Authors:** Vincent Loos, Rohit Pardasani, Navchetan Awasthi

**Affiliations:** a University of Amsterdam, Faculty of Science, Mathematics and Computer Science, Informatics Institute, Amsterdam, The Netherlands; b General Electric Healthcare, Bengaluru, Karnataka, India; c Amsterdam UMC, Department of Biomedical Engineering and Physics, Amsterdam, The Netherlands

**Keywords:** effective receptive field, receptive field, segmentation, theoretical receptive field, U-Net

## Abstract

**Purpose:**

Medical image segmentation is a critical task in healthcare applications, and U-Nets have demonstrated promising results in this domain. We delve into the understudied aspect of receptive field (RF) size and its impact on the U-Net and attention U-Net architectures used for medical imaging segmentation.

**Approach:**

We explore several critical elements including the relationship among RF size, characteristics of the region of interest, and model performance, as well as the balance between RF size and computational costs for U-Net and attention U-Net methods for different datasets. We also propose a mathematical notation for representing the theoretical receptive field (TRF) of a given layer in a network and propose two new metrics, namely, the effective receptive field (ERF) rate and the object rate, to quantify the fraction of significantly contributing pixels within the ERF against the TRF area and assessing the relative size of the segmentation object compared with the TRF size, respectively.

**Results:**

The results demonstrate that there exists an optimal TRF size that successfully strikes a balance between capturing a wider global context and maintaining computational efficiency, thereby optimizing model performance. Interestingly, a distinct correlation is observed between the data complexity and the required TRF size; segmentation based solely on contrast achieved peak performance even with smaller TRF sizes, whereas more complex segmentation tasks necessitated larger TRFs. Attention U-Net models consistently outperformed their U-Net counterparts, highlighting the value of attention mechanisms regardless of TRF size.

**Conclusions:**

These insights present an invaluable resource for developing more efficient U-Net-based architectures for medical imaging and pave the way for future exploration of other segmentation architectures. A tool is also developed, which calculates the TRF for a U-Net (and attention U-Net) model and also suggests an appropriate TRF size for a given model and dataset.

## 1 Introduction

Medical imaging, a cornerstone of modern healthcare, provides a non-invasive means for diagnosing and monitoring a wide range of diseases. However, the interpretation of medical images often requires expert knowledge and can be time-consuming, leading to a growing interest in automated analysis methods.^1^

**Fig. 1:**
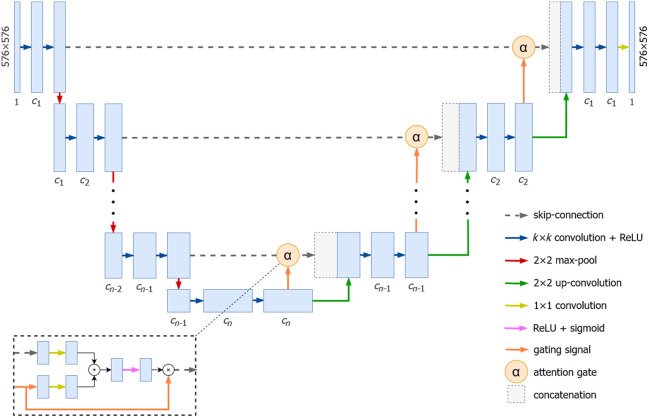
Variable attention U-Net in which the depth (n), kernel size of the convolution layers (k), and number of channels (c) can be tuned to alter the size of the TRF. It can be converted to a regular U-Net by simply removing the attention gates and gating signals.

Semantic segmentation, a key task in computer vision, plays a crucial role in this context. It involves the categorization of pixels in an image into predefined classes, enabling the delineation of anatomical structures and pathological regions in medical images.^2^ The U-Net architecture, a convolutional neural network (CNN) designed specifically for biomedical image segmentation, has emerged as a popular choice for semantic segmentation tasks in medical imaging.^3^ As illustrated in Fig. 1, it employs an encoder–decoder structure. The encoder progressively reduces the spatial dimensionality while increasing the feature representation, capturing the global context of the image. The decoder, on the other hand, gradually recovers the spatial information, enabling precise localization.^4^ The U-Net is renowned for its accuracy in semantic segmentation tasks.^3^ An extended version, the attention U-Net, integrates an attention mechanism to enhance feature capturing to improve overall performance.^5^

**Fig. 2:**
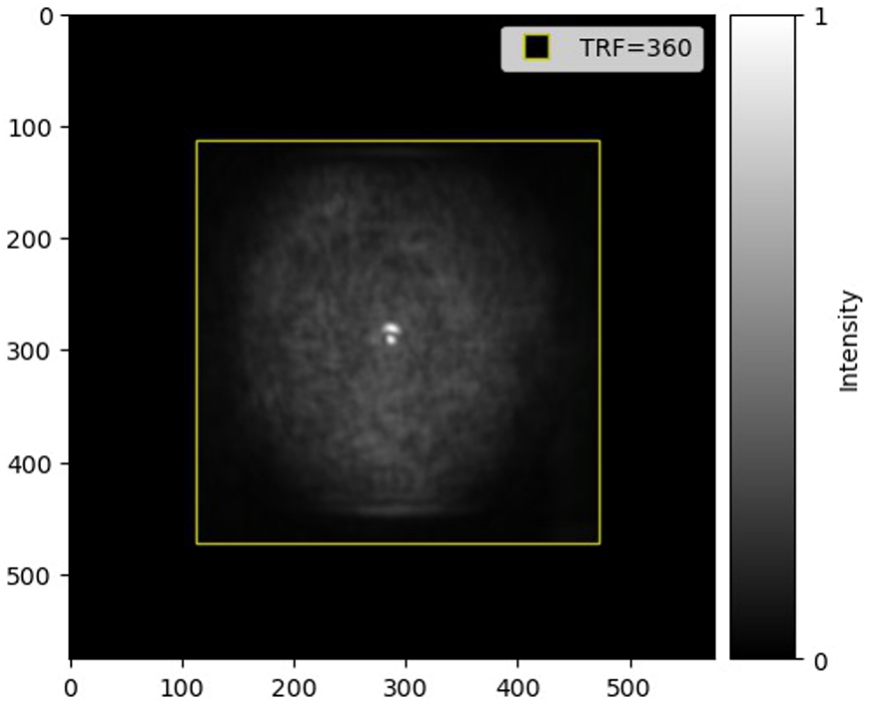
Example of TRF and ERF in an image. The yellow square denotes the TRF, the maximum input area influencing the output pixel located at the center of the square. The gray pixels, representing the ERF, show the actual input area affecting a neuron’s activation, with intensity indicating the impact level.

Within these networks, the concept of the receptive field (RF) is crucial. It refers to the region in the input space that affects a feature in a CNN.^6^ There are two kinds of RF: the theoretical receptive field (TRF) and the effective receptive field (ERF). The TRF is defined as the maximum region of the input image that influences a specific pixel of the output, considering only the RF from the preceding layers that are relevant to the current layer.^7^ This is in contrast to the ERF, which is the actual region of the input image that contributes to the activation of a particular neuron in the network, taking into account the impact of operations such as pooling.^6^ An example of the TRF and ERF is illustrated in Fig. 2.

Previous studies have started investigating the role of RF size on U-Net performance for image segmentation tasks but not all aspects have been explored. One study^8^ focused on ultrasound image segmentation, demonstrating that the RF size has a more critical role than the network’s depth or the number of parameters. They suggested that a computationally efficient shallow network could replace a deep one without performance loss by manipulating the RF size. However, their study was limited to the U-Net architecture and a single dataset, comparing only a deep and a shallow network, leaving room for a more exhaustive investigation.

Another study^9^ delved into the influence of RF size and network complexity on a CNN’s performance for transmission electron microscopy (TEM) image analysis. They found that the RF size’s influence varied with TEM image resolution and contrast characteristics. For low-resolution TEM images, where contrast is crucial, RF size had a minimal influence. However, for high-resolution TEM images, where identification is less dependent on contrast changes, the RF size was vital, especially for low-contrast images. However, they only considered TEM images and regular U-Nets, leaving the applicability of these findings to other medical imaging tasks unexplored.

Our study builds upon these insights by examining the influence of the RF size on U-Net and attention U-Net architectures across multiple medical image segmentation datasets with certain characteristics in the region of interest (RoI). We compare 10 different U-Net architectures shown in Table 1 with varied RF sizes and equal total parameters, thus isolating RF size’s impact performance while taking into consideration specific dataset characteristics. Moreover, we repeat the experiments on eight different synthetic datasets and six real-world medical datasets. We also extend our investigation to the attention U-Net architecture, thereby expanding the study beyond regular U-Net architecture. Our aim is to offer critical insights for U-Net-based architectures’ design, considering RF size as a key parameter.

**Table 1:** All U-Net configurations with different TRF sizes. The TRF size is influenced by the convolutional kernel size (k) and the vertical depth of the network (d).

TRF size	k,d	Out channels per layer	# Parameters
54	3, 2	[230, 456, 765, 1245]	31,013,720
100	3, 3	[145, 256, 512, 1024]	31,012,268
146	3, 4	[133, 244, 355, 791]	31,032,960
204	4, 3	[64, 128, 256, 512, 1024]	31,042,369
230	3, 6	[63, 170, 256, 512]	31,031,345
298	4, 4	[25, 44, 110, 451, 756]	31,043,816
360	3, 8	[47, 83, 180, 360]	31,062,482
412	5, 3	[63, 64, 115, 255, 512, 1024]	31,043,945
486	4, 6	[28, 58, 146, 270, 510]	31,027,119
570	4, 7	[24, 55, 101, 223, 481]	31,041,124

Specifically, this paper makes the following contributions to the field of medical image segmentation with U-Nets:

1. We provide a comprehensive analysis of the role of the RF size in the performance of U-Net and attention U-Net architectures, demonstrating its significance in capturing the necessary context for accurate segmentation.
2. We propose two new metrics called the ERF and object rates to quantify the fraction of significantly contributing pixels within the ERF against the TRF area and assessing the relative size of the segmentation object compared with the TRF size, respectively.
3. We explore the trade-off between RF size and computational cost for a variety of medical imaging and synthetic datasets.
4. We compare the performance of U-Net and attention U-Net architectures for the same RF size, highlighting the effectiveness of the attention mechanism in improving the model’s overall performance.
5. We present a nuanced analysis of the performance trends across datasets with different characteristics in the RoI, particularly its size and contrast to the surrounding area.
6. We provide a tool that calculates the TRF for a U-Net (and attention U-Net) model and also suggest an appropriate TRF size for a given model and dataset.

## 2 Methodology

This study explores the role of the RF in the performance of U-Net and attention U-Net models in semantic segmentation tasks. Through a series of experiments with varying TRF sizes, we evaluated these models on a diverse range of datasets. In this section, we further provide comprehensive descriptions of the model architectures, as well as the TRF and ERF computation.

### 2.1 U-Net Design and TRF Tuning

The configuration of the hyper-parameters of a U-Net model significantly impacts the size of its TRF. As established in previous works, two of the hyper-parameters that determine the TRF size in U-Net are the number of pooling layers and the convolutional kernel sizes.^10^ To elaborate, Fig. 1 illustrates a variable attention U-Net diagram, where the TRF size can be adjusted in two different ways. First, when the vertical depth (d) of the network is increased, one encoder and one decoder block are added before and after the bottleneck, respectively. This increases the number of pooling layers and therefore increases the TRF size. Changing the network depth on its own does not result in a significant impact on the model’s performance.^8^ Second, the TRF size can be varied by changing the kernel sizes of the convolutional layers within the network. The mathematical details of the effects of various layers on the TRF size are provided in Sec. 2.2.

It should be noted that adjusting these hyper-parameters also impacts the total number of parameters in the model. To ensure a fair comparison between the performance of various configurations, the total number of parameters must remain approximately equal. According to Ref. 11, this can be achieved by modifying the number of output channels in each convolution layer within the network blocks. Table 1 provides an overview of all configurations utilized in this study. It is important to mention that the parameter count is based on the standard U-Net architecture. The attention U-Net introduces additional parameters due to the inclusion of an attention block at each layer. However, this increase varies approximately on the order of 100,000, which is relatively insignificant and can be considered negligible in this context.

### 2.2 Computing the TRF

Formally, the TRF refers to the maximum region of the input image $X\in [0,1{]}^{h\times w}$ that potentially influences a specific pixel in the output layer. To represent the TRF at layer d in a U-Net architecture of depth D, we use a four-dimensional tensor $T^{\left(d\right)}\in R^{h\times w\times 2\times 2}$. Here, the first two dimensions correspond to the y and x axes of the input image, respectively, whereas the third and fourth dimensions represent the top-left (t-l) and bottom-right (b-r) coordinates of the TRF at layer d, respectively. For a given pixel located at position (i,j) in the output layer D, the TRF can be expressed as a 2×2 matrix in which the first row corresponds to the top-left corner of the TRF, and the second row corresponds to the bottom-right corner of the TRF

$$T_{i,j}^{\left(D\right)}=[\begin{array}{cc} t_{i,j}^{\left(D\right)} & l_{i,j}^{\left(D\right)}\\ b_{i,j}^{\left(D\right)} & r_{i,j}^{\left(D\right)} \end{array}].$$ (1)

Mathematically, it can be ascertained that all pixels have an equal TRF size in the output layer, except those located around the border because of the padded zeroes. Based on this observation, a single (maximum) TRF value can be assigned to the entire U-Net. In the remainder of this paper, we define the TRF size of a U-Net as the size of the TRF of the center pixel (u,v)=(h/2,w/2) in the output layer

$$\mathrm{TRF}=\sqrt{\left(T_{u,v,1,0}^{\left(D\right)}-T_{u,v,0,0}^{\left(D\right)})\cdot (T_{u,v,1,1}^{\left(D\right)}-T_{u,v,0,1}^{\left(D\right)}\right)}.$$ (2)

To compute the values of the TRF matrix in Eq. (1), we traverse the network from the first to the final layer, tracking the TRF of each pixel at every layer based on the previous layer’s pixels until reaching the output layer.^7^ Therefore, the TRF of the pixel at position (i,j) in a layer at depth d can be expressed as

$$T_{i,j}^{\left(d\right)}=[\begin{array}{cc} t_{i,j}^{\left(d\right)} & l_{i,j}^{\left(d\right)}\\ b_{i,j}^{\left(d\right)} & r_{i,j}^{\left(d\right)} \end{array}].$$ (3)

In the input layer 0, the TRF of each pixel corresponds to the pixel itself

$$T_{i,j}^{\left(0\right)}=[\begin{array}{cc} i & j\\ i & j \end{array}].$$ (4)

The computation of the TRF in subsequent layers depends on the U-Net’s configuration. The computation of TRF for all the possible layers, blocks, and functions that a U-Net may include is shown in Appendices A–F.

### 2.3 Computing the ERF

For each pixel $x_{i,j}$ in the input image $X\in [0,1{]}^{h\times w}$, its impact on the center pixel of the output image $y_{h/2,w/2}$ is measured by computing the partial derivative of the center output pixel with respect to each input pixel $\partial y_{h/2,w/2}/\partial x_{i,j}$. This method quantifies how much $y_{h/2,w/2}$ changes if $x_{i,j}$ is changed by a small amount.^6^ For a TRF, the corresponding ERF ($E\in R^{m\times n}$) can be expressed as a matrix, as shown in Eq. (5):

$$E=[\begin{array}{ccc} \frac{\partial y_{h/2,w/2}}{\partial x_{t,l}} & \ldots & \frac{\partial y_{h/2,w/2}}{\partial x_{t,r}}\\ \vdots & \ddots & \vdots \\ \frac{\partial y_{h/2,w/2}}{\partial x_{b,l}} & \ldots & \frac{\partial y_{h/2,w/2}}{\partial x_{b,r}} \end{array}].$$ (5)

The actual computation of the ERF can be done easily with most deep learning frameworks by back-propagating the value of one certain output pixel to the entire input and taking the m×n slice of the input at the position of the TRF.

## 3 Experiment

### 3.1 Training Protocol

All the models were trained on a high-performance computing node featuring two Intel Xeon Platinum 8360Y CPUs and an NVIDIA A100 GPU with 40 GB of HBM2 memory. We used the PyTorch framework^12^ and employed binary cross-entropy with logits loss as our loss function, with the Adam optimizer facilitating training due to its efficiency and minimal memory requirements.^13^

The initial learning rate was set at $10^{-4}$, and a learning rate scheduling strategy was implemented to optimize learning. This strategy reduces the learning rate by 0.1 when the validation loss plateaus for four epochs, enabling more substantial updates in early training phases and smaller updates as the model nears convergence. The training lasted up to 200 epochs, with early stopping^14^ implemented to prevent overfitting. If the validation loss remained static over 20 consecutive epochs, training was ceased, and the parameters that achieved the lowest validation loss were saved.

### 3.2 Datasets

**Fig. 3:**
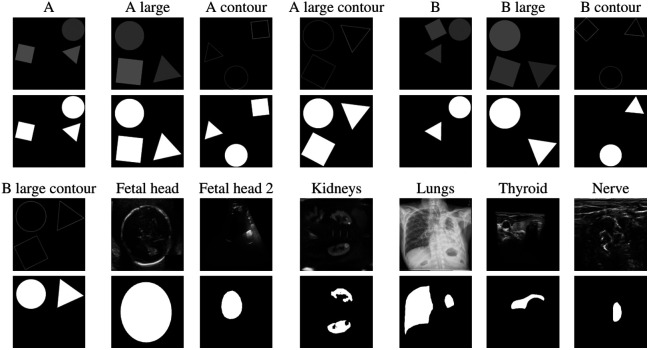
Typical images and segmentation masks for the synthetic datasets (A and B) and medical datasets (fetal head, fetal head 2, kidneys, lungs, thyroid, and nerve).

Our study utilized a wide array of datasets, both synthetic and real-world medical images. The synthetic datasets were specifically designed to evaluate certain hypotheses under controlled conditions. Following this, we applied our hypotheses to medical imaging datasets, which encompassed a variety of imaging techniques and anatomical structures, adding a layer of complexity and realism to our evaluations. Illustrative examples of images and corresponding masks from each dataset can be found in Fig. 3.

#### 3.2.1 Synthetic datasets

The synthetic shape datasets are designed to provide a controlled environment for investigating the impact of the TRF on the performance of the models. The datasets consist of synthetic images with predefined shapes and configurations, allowing for a systematic exploration of the models’ behavior under different conditions.

There are a total of eight datasets with generated images. These are of two types, referred to as type A and type B. Both types include three non-overlapping shapes—a circle, a triangle, and a square—that are randomly placed and rotated, with a random gray value assigned to each shape. For type A images, the masks are identical to the shapes in the images. For type B images, the masks are the same, but the mask of the square is omitted, adding an additional level of complexity to the segmentation task.

For each type, four datasets are created. Two of them contain small shapes placed on an invisible 3×3 grid, and two of them contain large shapes placed on an invisible 2×2 grid. For both the small and large datasets, one of them contains images with filled shapes and filled masks, and the other one contains images with only the contours of the shapes with filled masks. Each dataset contains a total of 1000 images, of which 700 are used for training, 150 for validation, and 150 for testing.

These synthetic shape datasets offer valuable insights into the role of the TRF on the performance of the models. By comparing the performance of the models on images with small shapes versus large shapes, we can assess how the TRF size affects the model’s ability to capture features of different scales. Specifically, it allows us to determine to what degree it matters if the TRF is smaller than the shape, or if the shape fits into the TRF.

The comparison between images with filled shapes and those with contour shapes allows us to determine what happens if the TRF does not capture the entire shape, but only a part of it, such as the part that is completely black in the image but is filled in the mask because it is within the contours. This is particularly relevant for real-world applications, where the images often contain complex structures that the model needs to accurately segment.

Furthermore, the use of type B images, where the mask of the square is omitted, enables us to examine how the models handle irrelevant features in the images. This is particularly relevant for real-world applications, where the images often contain irrelevant or distracting features that the model needs to ignore to perform the task effectively.

#### 3.2.2 Medical datasets

The experiments were carried out using below listed six medical datasets. The datasets are classified into two categories: high contrast, where the RoI can be visually distinguished solely based on its contrast with the background, and low contrast, which requires additional details such as the RoI’s contour or shape to distinguish it from the background.

1. Fetal head: This low-contrast dataset consists of two-dimensional (2D) ultrasound images of fetal heads (dataset).^15^ It includes 350 training images, 74 validation images, and 76 test images. The images were obtained using a standard clinical ultrasound system, and the fetal head circumference was manually annotated by expert sonographers.
2. Fetal head 2: This low-contrast dataset is another set of 2D ultrasound images of fetal heads, with a larger number of images (dataset).^16^^,^^17^ It includes 14,560 training images, 3240 validation images, and 2875 test images. The images in this dataset were collected from multiple hospitals and were annotated by experienced radiologists.
3. Kidneys: This low-contrast dataset consists of three-dimensional (3D) MRI images of kidneys (dataset).^18^^,^^19^ It includes 454 training images, 91 validation images, and 104 test images. The images were acquired using a 3T MRI scanner, and the kidney regions were manually segmented by radiologists.
4. Lungs: This high-contrast dataset consists of 2D X-ray images of lungs (dataset).^20^^,^^21^ It includes 396 training images, 84 validation images, and 86 test images. The images were collected from a variety of patients with different lung conditions, providing a diverse dataset for training and testing.
5. Thyroid: This low-contrast dataset consists of 3D ultrasound images of the thyroid (dataset).^22^ It includes 3160 training images, 439 validation images, and 510 test images. The images were acquired using a high-frequency linear array transducer, and the thyroid regions were manually segmented by experienced clinicians.
6. Nerve: This low-contrast dataset consists of 2D ultrasound images of nerves (dataset).^23^ It includes 1610 training images, 364 validation images, and 349 test images. The images were collected from a variety of patients, and the nerve structures were manually annotated by expert radiologists.

### 3.3 Data Pre-processing

All images in the datasets were pre-processed to ensure consistency and optimal performance of the models. The pre-processing steps included resizing all images to a uniform size of 576×576  pixels. For the 3D datasets, all 2D slices were extracted and used as separate images.

The datasets were split into training, validation, and test sets, with ∼70% of the images used for training, 15% for validation, and 15% for testing. However, to prevent overfitting, slices from one 3D volume or 2D images from the same patient were included in only one of the train, validation, or test sets. This means that the split is not always exactly in these ratios.

Finally, on some of the smaller datasets, random data augmentation was applied to improve the absolute results. On each sample, four random combinations of a horizontal flip, vertical flip, and rotation with 90, 180, or 270 deg, respectively, were applied, where each part of the combination is applied with a probability of 0.5.

### 3.4 Evaluation Measures

In the realm of image segmentation, five principal metrics are typically utilized to assess performance.^24^^,^^25^ The Dice similarity coefficient (DSC) serves as a statistical metric, measuring the similarity between two sets by calculating the ratio of twice the intersection area to the total size of both sets. Sensitivity, or recall, appraises the model’s ability to accurately identify positive instances, hence providing insight into the model’s efficacy in segmenting intended areas. Specificity evaluates the model’s proficiency in correctly recognizing negative instances, or in other words, its capability to exclude regions not meant to be segmented. Accuracy gauges the model’s overall correctness in assigning classifications. Last, the Jaccard index (JI) is an intersection-over-union measure that quantifies the similarity between the predicted and actual segmentations, providing a rigorous assessment of model performance in segmenting images.

Moreover, to understand fully the impact of TRF and ERF on model performance, two additional metrics are proposed in this work: ERF rate and object rate. We also factor in the training time (epochs) as a metric, quantifying the epochs needed to attain the lowest validation loss. This allows us to comparatively analyze the training cost across various models.

#### 3.4.1 ERF rate

We propose a new metric called the ERF rate to measure the ERF distribution. It quantifies the fraction of significantly contributing pixels within the ERF against the TRF area, utilizing the absolute value of the ERF pixels. The ERF rate [Eq. (6)] accounts for all the meaningful pixels above a certain threshold (ε) in the ERF, giving more weight to pixels with higher values and normalizing the result with the TRF area. The metric is computed for each test image, reporting the mean ERF rate as the overall score. Thus, ERF rate calculation is tightly coupled to the dataset as the weights and input images contributing to the calculation of ERF are associated with the dataset

$$r=\frac{\underset{y\in E}{\sum }[|y|>\varepsilon ]\cdot (1+|y|)}{m\cdot n}.$$ (6)

We use kernel density estimation (KDE) to find the threshold (ε) for key contributing pixels, estimating the probability density function (PDF) of a continuous variable based on observed samples.^26^

The density function f(x) of ERF values can be calculated using Eq. (7), where $\hat{f}(x)$ is the estimated PDF, K(x) is a kernel function with bandwidth h, and m·n is the number of observations in E. It is centered on each observation y

f^(x)=1m·n·∑y∈E1hK(x−|y|h). (7)

To identify the ideal parameters for KDE, we examined the ERF absolute value histogram for a large dataset sample. It reveals two different types of distributions: (i) ERFs with both contributing and non-contributing pixels have a bimodal distribution with a left peak representing non-contributing pixels and a right peak representing contributing pixels, and (ii) ERFs with mostly non-contributing pixels have a highly positively skewed distribution. The first parameter, the bandwidth (h), controls the kernel width and PDF smoothing level. Silverman’s rule-of-thumb^27^ was used to automatically determine h (h=1.06·σ^mn, where $\hat{\sigma }$ is the standard deviation of a sample of size m·n) because it performs well on both bimodal and skewed distributions.^28^

**Fig. 4.**
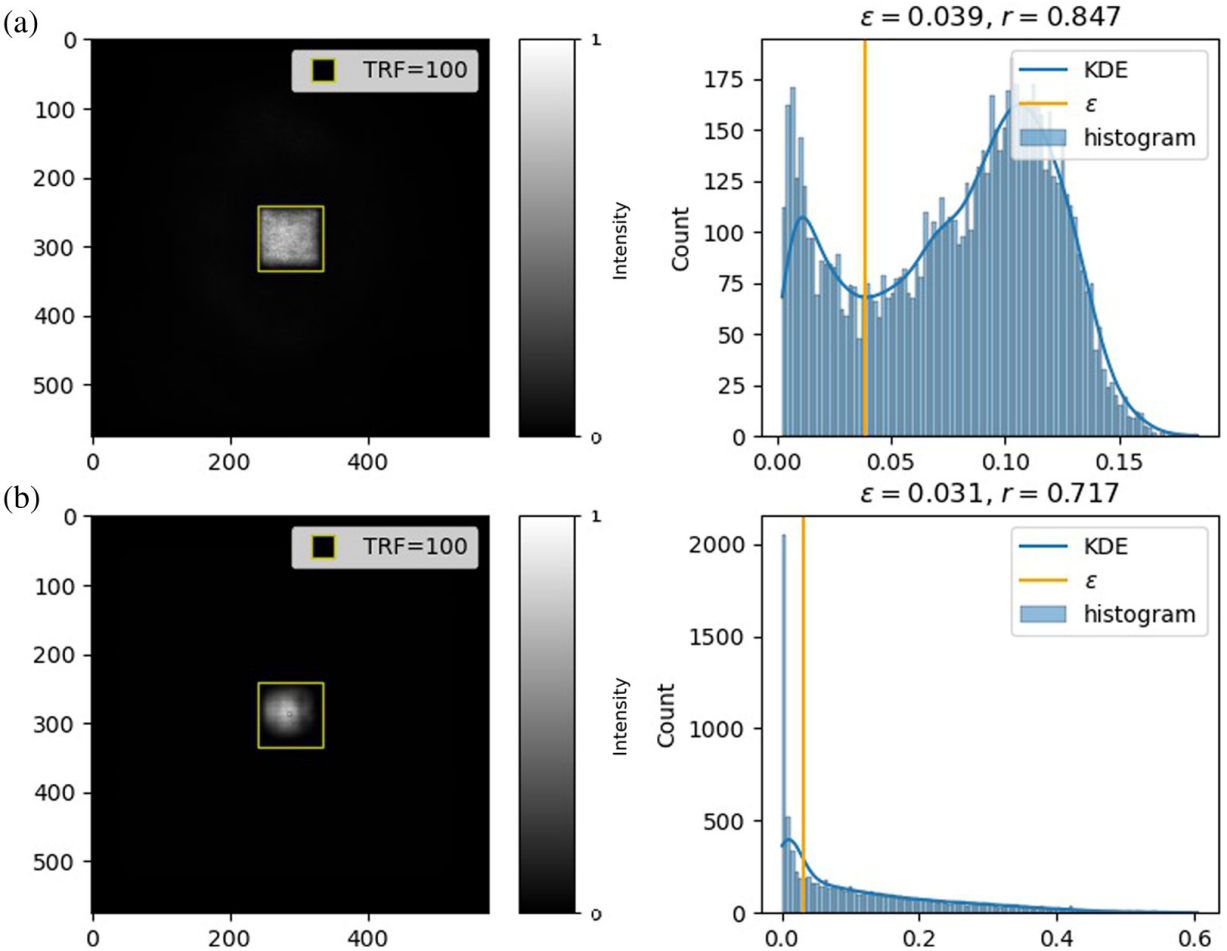
Examples of determining the threshold (ε) for the ERF rate with KDE for bimodally distributed ERF pixel values (a) and positively skewed distributed ERF pixel values (b).

Finally, the threshold (ε) was selected based on the trough in bimodal distributions or the stopping point of decrease in skewed distributions (Fig. 4). To reduce the number of troughs, thus making it easier to find the optimal threshold, a Gaussian kernel function was used to smooth the estimated PDF.^26^ The empirical formula for the stopping point amounts to finding a point in the histogram where the frequency value in the bin is below the mean of the frequency values in the neighboring bins. This is approximately equal to numerically finding a point where the histogram curve becomes convex.

The ERF rate intends to assess the utilization of TRF. A low ERF rate mathematically signifies that pixels contributing to the output are more localized as compared to the region being explored through TRF. Mathematically, a low ERF rate should signal the under-utilization of TRF, whereas an ERF rate close (or above 1) would indicate that TRF size might not be enough to learn all the relevant features of the dataset.

#### 3.4.2 Object rate

To assess the relative size of the object to be segmented in comparison to the TRF size, a new metric denoted as the object rate is proposed. This metric is computed by dividing the total area of a rectangle encompassing the edges of the object by the total area of the TRF size, or ${\mathrm{TRF}}^{2}$ as defined in Eq. (2). In the case of multiple objects, the bounding box of each object is considered individually, and the average area of such rectangles is computed. Thus, for an object with its highest point at t, lowest at b, leftmost at l, and rightmost at r, the object rate can be calculated as follows:

$$\mathrm{OR}=\frac{\left(b-t)\cdot (r-l\right)}{{\mathrm{TRF}}^{2}}.$$ (8)

## 4 Results and Discussion

Detailed results of the performance of the U-Net model for the different metrics on all medical datasets can be found in Table 2. The results of the attention U-Net on the medical datasets and the U-Net on the synthetic datasets of types A and B can be found in Tables 3–5, respectively. In Secs. 4.1, 4.2 and 4.3 we present different plots to interpret and discuss these results.

**Table 2:** All the results for the different evaluation measures on the medical datasets (fetal head, fetal head 2, kidneys, lungs, nerve, and thyroid) for the U-Net.

TRF size	trf54	trf100	trf146	trf204	trf230	trf298	trf360	trf412	trf486	trf570
Fetal head										
Training time (epochs)	38	31	21	26	34	21	24	26	28	30
ERF rate before training	0.0135	0.0097	0.0047	0.0011	0.0046	0.0014	0.0044	0.0005	0.0012	0.0009
ERF rate	0.8898	0.9380	0.9153	0.8614	0.8196	0.5970	0.5785	0.4618	0.4175	0.2309
Dice score	0.7752	0.8866	0.9224	0.9527	0.9506	0.9526	0.9623	0.9614	0.9650	0.9665
Object rate	67.3558	16.8389	7.4840	4.0325	2.6942	1.7922	1.0997	0.9870	0.6452	0.4481
Accuracy	0.8687	0.9300	0.9518	0.9690	0.9693	0.9704	0.9749	0.9735	0.9761	0.9773
Sensitivity	0.8458	0.8914	0.9395	0.9508	0.9707	0.9709	0.9675	0.9570	0.9627	0.9680
Specificity	0.8831	0.9530	0.9612	0.9828	0.9739	0.9745	0.9844	0.9878	0.9887	0.9873
JI	0.6577	0.8152	0.8723	0.9212	0.9210	0.9248	0.9390	0.9371	0.9435	0.9466
Fetal head 2										
Training time (epochs)	1	1	1	6	1	8	7	7	25	14
ERF rate before training	0.1531	0.0330	0.0300	0.0097	0.0245	0.0073	0.0163	0.0019	0.0051	0.0054
ERF rate	1.0005	0.0944	0.2275	0.0095	0.0168	0.0051	0.2761	0.0010	0.3159	0.1735
Dice score	0.6009	0.6261	0.7582	0.8745	0.7950	0.8588	0.9028	0.9071	0.9116	0.9214
Object rate	35.5599	8.8900	3.9511	2.1289	1.4224	0.9462	0.5806	0.5211	0.3406	0.2365
Accuracy	0.9889	0.8888	0.9207	0.9557	0.9315	0.9525	0.9654	0.9675	0.9686	0.9725
Sensitivity	0.7058	0.7818	0.8512	0.9133	0.9284	0.9502	0.9567	0.9524	0.9479	0.9616
Specificity	0.9913	0.9004	0.9303	0.9627	0.9316	0.9514	0.9645	0.9679	0.9708	0.9723
Jaccard index	0.5154	0.4764	0.6265	0.7876	0.6841	0.7805	0.8365	0.8445	0.8513	0.8656
Kidneys										
Training time (epochs)	21	34	31	32	38	47	54	47	48	54
ERF rate before training	0.1715	0.0496	0.0303	0.0126	0.0287	0.0058	0.0217	0.0024	0.0059	0.0065
ERF rate	0.0123	0.0341	0.0227	0.0057	0.0162	0.0035	0.0088	0.0012	0.0038	0.0038
Dice score	0.7560	0.8367	0.8477	0.8524	0.8617	0.8364	0.8865	0.8657	0.8439	0.8802
Object rate	27.4954	6.8738	3.0550	1.6461	1.0998	0.7316	0.4489	0.4029	0.2634	0.1829
Accuracy	0.9832	0.9904	0.9911	0.9911	0.9917	0.9889	0.9923	0.9902	0.9900	0.9918
Sensitivity	0.7808	0.8856	0.8762	0.8783	0.8645	0.8814	0.8993	0.8914	0.8647	0.8884
Specificity	0.9892	0.9935	0.9945	0.9948	0.9963	0.9916	0.9961	0.9935	0.9942	0.9954
Jaccard index	0.6320	0.7509	0.7679	0.7717	0.7853	0.7490	0.8119	0.7836	0.7607	0.8055
Lungs										
Training time (epochs)	15	20	29	26	36	26	51	28	33	40
ERF rate before training	0.0417	0.0419	0.0216	0.0127	0.0122	0.0069	0.0134	0.0009	0.0036	0.0061
ERF rate	0.0614	0.1298	0.0315	0.0040	0.0137	0.0031	0.0245	0.0012	0.0011	0.0039
Dice score	0.9601	0.9673	0.9687	0.9686	0.9683	0.9666	0.9689	0.9683	0.9662	0.9673
Object rate	84.4219	21.1055	9.3802	5.0542	3.3769	2.2463	1.3784	1.2371	0.8087	0.5616
Accuracy	0.9784	0.9823	0.9830	0.9829	0.9829	0.9820	0.9830	0.9827	0.9818	0.9824
Sensitivity	0.9650	0.9677	0.9697	0.9681	0.9695	0.9654	0.9694	0.9654	0.9746	0.9776
Specificity	0.9825	0.9870	0.9872	0.9878	0.9869	0.9873	0.9875	0.9886	0.9834	0.9834
Jaccard index	0.9240	0.9371	0.9398	0.9396	0.9391	0.9361	0.9402	0.9389	0.9353	0.9373
Nerve										
Training time (epochs)	7	13	15	8	14	17	10	8	10	12
ERF rate before training	0.1425	0.0444	0.0381	0.0107	0.0287	0.0096	0.0213	0.0019	0.0104	0.0078
ERF rate	0.9312	0.7345	0.6953	0.0057	0.3348	0.0224	0.1363	0.0008	0.1244	0.0399
Dice score	0.4685	0.7329	0.7531	0.7745	0.7792	0.7863	0.7965	0.7951	0.7960	0.7947
Object rate	7.3183	1.8296	0.8131	0.4381	0.2927	0.1947	0.1195	0.1072	0.0701	0.0487
Accuracy	0.9758	0.9848	0.9859	0.9868	0.9873	0.9872	0.9876	0.9878	0.9881	0.9880
Sensitivity	0.6442	0.7519	0.7637	0.7803	0.7978	0.7990	0.7901	0.8068	0.8301	0.8289
Specificity	0.9808	0.9914	0.9923	0.9930	0.9928	0.9930	0.9940	0.9934	0.9927	0.9927
Jaccard index	0.3281	0.6030	0.6321	0.6572	0.6614	0.6701	0.6827	0.6800	0.6786	0.6785
Thyroid										
Training time (epochs)	1	1	3	2	2	5	3	2	7	4
ERF rate before training	0.1652	0.0434	0.0280	0.0094	0.0227	0.0093	0.0180	0.0021	0.0089	0.0054
ERF rate	0.1041	0.1439	0.1823	0.0124	0.0482	0.0240	0.0269	0.0038	0.0152	0.0054
Dice score	0.5155	0.5829	0.6456	0.7043	0.7124	0.6907	0.6680	0.6667	0.7457	0.7284
Object rate	14.8609	3.7152	1.6512	0.8897	0.5944	0.3954	0.2426	0.2178	0.1424	0.0989
Accuracy	0.9718	0.9807	0.9840	0.9860	0.9822	0.9864	0.9854	0.9859	0.9871	0.9837
Sensitivity	0.6309	0.7427	0.7374	0.7516	0.7021	0.7563	0.7449	0.7705	0.7602	0.7268
Specificity	0.9788	0.9827	0.9879	0.9912	0.9928	0.9905	0.9884	0.9879	0.9935	0.9933
Jaccard index	0.3881	0.4779	0.5481	0.6107	0.6168	0.6036	0.5785	0.5746	0.6526	0.6246

**Table 3:** All results for the different evaluation measures on the medical datasets (fetal head, fetal head 2, kidneys, lungs, nerve, and thyroid) for the Attention- U-Net.

TRF size	trf54	trf100	trf146	trf204	trf230	trf298	trf360	trf412	trf486	trf570
Fetal head										
Training time (epochs)	36	28	26	34	35	39	39	34	32	34
ERF rate before training	0.8256	0.5080	0.4090	0.4592	0.3215	0.3601	0.2955	0.4388	0.2972	0.2690
ERF rate	0.9854	0.9548	0.9732	0.1771	0.8143	0.0024	0.5025	0.0013	0.0016	0.0025
Dice score	0.8307	0.9213	0.9524	0.9538	0.9625	0.9640	0.9642	0.9655	0.9675	0.9667
Object rate	67.3558	16.8389	7.4840	4.0325	2.6942	1.7922	1.0997	0.9870	0.6452	0.4481
Accuracy	0.8984	0.9512	0.9708	0.9715	0.9769	0.9781	0.9780	0.9791	0.9807	0.9803
Sensitivity	0.9085	0.9553	0.9528	0.9727	0.9591	0.9665	0.9637	0.9621	0.9638	0.9671
Specificity	0.8920	0.9476	0.9799	0.9711	0.9869	0.9848	0.9865	0.9891	0.9905	0.9879
Jaccard index	0.7200	0.8656	0.9206	0.9231	0.9388	0.9417	0.9420	0.9445	0.9482	0.9469
Fetal head 2										
Training time (epochs)	6	3	4	2	4	8	4	6	4	12
ERF rate before training	0.8459	0.5388	0.4327	0.5030	0.3511	0.4051	0.3217	0.4826	0.3293	0.3049
ERF rate	1.0000	0.9070	0.8755	0.0325	0.7660	0.0845	0.3467	0.0030	0.1717	0.1735
Dice score	0.7094	0.8058	0.8403	0.8574	0.8895	0.9025	0.9082	0.9135	0.9106	0.9224
Object rate	35.5599	8.8900	3.9511	2.1289	1.4224	0.9462	0.5806	0.5211	0.3406	0.2850
Accuracy	0.9155	0.9380	0.9476	0.9508	0.9621	0.9658	0.9673	0.9699	0.9698	0.9518
Sensitivity	0.8614	0.8797	0.9172	0.9281	0.9419	0.9521	0.9465	0.9424	0.9513	0.9395
Specificity	0.9232	0.9479	0.9516	0.9529	0.9648	0.9668	0.9688	0.9739	0.9705	0.9612
Jaccard index	0.5716	0.6892	0.7341	0.7616	0.8130	0.8350	0.8441	0.8542	0.8517	0.8723
Kidneys										
Training time (epochs)	27	29	25	48	52	50	57	57	65	55
ERF rate before training	0.8694	0.5124	0.4018	0.4715	0.3262	0.3916	0.2979	0.4270	0.3122	0.2975
ERF rate	0.1832	0.1251	0.0902	0.0406	0.1925	0.0388	0.0122	0.0015	0.0049	0.0019
Dice score	0.7481	0.8529	0.8410	0.8542	0.8709	0.8484	0.8703	0.8558	0.8979	0.8586
Object rate	27.4954	6.8738	3.0550	1.6461	1.0998	0.7316	0.4489	0.4029	0.2634	0.1829
Accuracy	0.9829	0.9911	0.9896	0.9911	0.9917	0.9901	0.9918	0.9913	0.9930	0.9903
Sensitivity	0.8210	0.8484	0.8500	0.8813	0.8878	0.8360	0.8813	0.8849	0.8920	0.8742
Specificity	0.9867	0.9959	0.9951	0.9942	0.9951	0.9952	0.9954	0.9952	0.9965	0.9936
Jaccard index	0.6197	0.7720	0.7505	0.7747	0.7967	0.7687	0.7990	0.7798	0.8300	0.7759
Lungs										
Training time (epochs)	21	22	30	16	31	23	29	17	30	30
ERF rate before training	0.8377	0.5446	0.4295	0.5266	0.3453	0.3745	0.3063	0.4558	0.3053	0.3143
ERF rate	0.8615	0.6073	0.4391	0.0071	0.0240	0.1075	0.0245	0.0044	0.0084	0.0045
Dice score	0.9574	0.9677	0.9673	0.9672	0.9668	0.9662	0.9671	0.9649	0.9666	0.9681
Object rate	84.4219	21.1055	9.3802	5.0542	3.3769	2.2463	1.3784	1.2371	0.8087	0.5616
Accuracy	0.9769	0.9824	0.9822	0.9823	0.9819	0.9817	0.9822	0.9810	0.9819	0.9827
Sensitivity	0.9488	0.9738	0.9629	0.9665	0.9728	0.9636	0.9711	0.9618	0.9685	0.9684
Specificity	0.9867	0.9850	0.9886	0.9872	0.9846	0.9875	0.9856	0.9871	0.9862	0.9874
Jaccard index	0.9191	0.9378	0.9371	0.9370	0.9363	0.9352	0.9370	0.9331	0.9362	0.9388
Nerve										
Training time (epochs)	10	12	12	13	10	10	14	9	14	11
ERF rate before training	0.8478	0.5326	0.4261	0.4985	0.3459	0.3965	0.3144	0.4639	0.3263	0.3110
ERF rate	0.9638	0.8183	0.8422	0.4236	0.5661	0.2254	0.4018	0.0762	0.2615	0.1329
Dice score	0.4801	0.7014	0.7428	0.7708	0.7631	0.7689	0.7911	0.7911	0.7941	0.7964
Object rate	7.3183	1.8296	0.8131	0.4381	0.2927	0.1947	0.1195	0.1072	0.0701	0.0487
Accuracy	0.9746	0.9849	0.9860	0.9872	0.9869	0.9867	0.9881	0.9875	0.9880	0.9881
Sensitivity	0.5738	0.8122	0.7848	0.7959	0.8150	0.7957	0.8210	0.8139	0.8343	0.8228
Specificity	0.9824	0.9885	0.9911	0.9924	0.9914	0.9922	0.9927	0.9929	0.9924	0.9930
Jaccard index	0.3427	0.5687	0.6203	0.6541	0.6437	0.6490	0.6761	0.6765	0.6770	0.6805
Thyroid										
Training time (epochs)	2	2	2	3	4	3	3	2	3	5
ERF rate before training	0.8760	0.5268	0.4179	0.4842	0.3426	0.4035	0.3087	0.4485	0.2973	0.3025
ERF rate	0.8913	0.6137	0.3449	0.0786	0.1057	0.0504	0.1024	0.0125	0.0468	0.0253
Dice score	0.5706	0.6773	0.6638	0.7464	0.7142	0.7181	0.7060	0.7455	0.7219	0.7420
Object rate	14.8609	3.7152	1.6512	0.8897	0.5944	0.3954	0.2426	0.2178	0.1424	0.0989
Accuracy	0.9704	0.9811	0.9819	0.9844	0.9818	0.9835	0.9839	0.9832	0.9802	0.9836
Sensitivity	0.6093	0.7146	0.7767	0.7933	0.7155	0.7677	0.7581	0.7960	0.7262	0.7863
Specificity	0.9832	0.9897	0.9871	0.9904	0.9914	0.9903	0.9899	0.9902	0.9922	0.9901
Jaccard index	0.4402	0.5736	0.5609	0.6372	0.6085	0.6137	0.6084	0.6343	0.6114	0.6333

**Table 4:** Results of the regular U-Net model on the Type A shapes datasets.

TRF size	54	100	146	204	230	298	360	412	486	570
A										
Training time (epochs)	63	66	111	71	69	71	200	74	192	72
ERF rate before training	0.0514	0.0131	0.0104	0.0033	0.0078	0.0043	0.0080	0.0005	0.0015	0.0044
ERF rate	0.0028	0.0527	0.0009	0.0008	0.0008	0.0006	0.0002	0.0002	0.0002	0.0002
Dice score	1.0000	1.0000	1.0000	1.0000	1.0000	1.0000	1.0000	1.0000	1.0000	1.0000
Object rate	100.5928	25.1482	11.1770	6.0224	4.0237	2.6766	1.6424	1.4741	0.9636	0.6692
Accuracy	1.0000	1.0000	1.0000	1.0000	1.0000	1.0000	1.0000	1.0000	1.0000	1.0000
Sensitivity	1.0000	1.0000	1.0000	1.0000	1.0000	1.0000	1.0000	1.0000	1.0000	1.0000
Specificity	1.0000	1.0000	1.0000	1.0000	1.0000	1.0000	1.0000	1.0000	1.0000	1.0000
Jaccard index	1.0000	1.0000	1.0000	1.0000	1.0000	1.0000	1.0000	1.0000	1.0000	1.0000
A contour										
Training time (epochs)	14	17	87	18	200	47	200	39	49	117
ERF rate before training	0.0353	0.0138	0.0088	0.0038	0.0059	0.0020	0.0040	0.0004	0.0010	0.0011
ERF rate	0.4710	0.0502	0.0392	0.0048	0.0047	0.0029	0.0036	0.0005	0.0013	0.0017
Dice score	0.8219	0.9791	0.9998	0.9996	0.9996	0.9997	0.9998	0.9996	0.9997	0.9999
Object rate	98.8970	24.7242	10.9886	5.9208	3.9559	2.6315	1.6147	1.4492	0.9473	0.6579
Accuracy	0.9469	0.9930	0.9999	0.9999	0.9999	0.9999	0.9999	0.9999	0.9999	1.0000
Sensitivity	0.9505	0.9933	0.9999	0.9997	0.9996	0.9998	0.9998	0.9997	0.9997	0.9999
Specificity	0.9465	0.9929	1.0000	0.9999	0.9999	0.9999	1.0000	0.9999	0.9999	1.0000
Jaccard index	0.6983	0.9591	0.9996	0.9992	0.9992	0.9995	0.9997	0.9992	0.9994	0.9997
A large										
Training time (epochs)	50	64	57	70	97	68	105	67	99	69
ERF rate before training	0.0326	0.0097	0.0115	0.0019	0.0058	0.0008	0.0047	0.0008	0.0020	0.0015
ERF rate	0.0028	0.0007	0.0004	0.0003	0.0002	0.0002	0.0001	0.0001	0.0001	0.0000
Dice score	1.0000	1.0000	1.0000	1.0000	1.0000	1.0000	1.0000	1.0000	1.0000	1.0000
Object rate	137.8993	34.4748	15.3221	8.2559	5.5160	3.6693	2.2515	2.0207	1.3209	0.9173
Accuracy	1.0000	1.0000	1.0000	1.0000	1.0000	1.0000	1.0000	1.0000	1.0000	1.0000
Sensitivity	1.0000	1.0000	1.0000	1.0000	1.0000	1.0000	1.0000	1.0000	1.0000	1.0000
Specificity	1.0000	1.0000	1.0000	1.0000	1.0000	1.0000	1.0000	1.0000	1.0000	1.0000
Jaccard index	1.0000	1.0000	1.0000	1.0000	1.0000	1.0000	1.0000	1.0000	1.0000	1.0000
A large contour										
Training time (epochs)	25	31	56	13	24	199	19	12	25	33
ERF rate before training	0.0290	0.0148	0.0081	0.0037	0.0055	0.0031	0.0052	0.0006	0.0013	0.0014
ERF rate	0.4730	0.2601	0.8689	0.0028	0.0034	0.0015	0.0025	0.0003	0.0006	0.0007
Dice score	0.6708	0.8646	0.9841	0.9995	0.9997	0.9993	0.9998	0.9995	0.9994	0.9994
Object rate	138.7451	34.6863	15.4161	8.3065	5.5498	3.6918	2.2653	2.0331	1.3290	0.9229
Accuracy	0.8197	0.9119	0.9892	0.9997	0.9998	0.9995	0.9998	0.9997	0.9996	0.9996
Sensitivity	0.8949	0.9112	0.9912	0.9995	0.9997	0.9993	0.9998	0.9995	0.9995	0.9993
Specificity	0.8005	0.9123	0.9881	0.9998	0.9998	0.9996	0.9999	0.9998	0.9996	0.9998
Jaccard index	0.5056	0.7626	0.9688	0.9991	0.9994	0.9986	0.9995	0.9990	0.9987	0.9989

**Table 5:** Results of the regular U-Net model on the Type B shapes datasets.

TRF size	54	100	146	204	230	298	360	412	486	570
B										
Training time (epochs)	46	52	173	190	123	47	45	44	45	198
ERF rate before training	0.0387	0.0113	0.0128	0.0022	0.0105	0.0022	0.0054	0.0007	0.0019	0.0019
ERF rate	0.0918	0.0556	0.0016	0.0010	0.0005	0.0006	0.0002	0.0002	0.0001	0.0001
Dice score	0.9345	0.9959	1.0000	1.0000	1.0000	1.0000	0.9998	1.0000	0.9999	1.0000
Object rate	57.3475	14.3369	6.3719	3.4333	2.2939	1.5259	0.9363	0.8404	0.5493	0.3815
Accuracy	0.9843	0.9991	1.0000	1.0000	1.0000	1.0000	1.0000	1.0000	1.0000	1.0000
Sensitivity	0.8810	0.9921	1.0000	1.0000	1.0000	0.9999	0.9998	1.0000	0.9999	1.0000
Specificity	0.9994	1.0000	1.0000	1.0000	1.0000	1.0000	1.0000	1.0000	1.0000	1.0000
Jaccard index	0.8772	0.9920	1.0000	1.0000	1.0000	0.9999	0.9996	1.0000	0.9999	1.0000
B contour										
Training time (epochs)	20	12	81	125	137	42	194	139	144	182
ERF rate before training	0.0277	0.0063	0.0059	0.0018	0.0061	0.0019	0.0046	0.0005	0.0013	0.0012
ERF rate	0.5588	0.3507	0.0383	0.0018	0.0042	0.0012	0.0033	0.0006	0.0022	0.0009
Dice score	0.8067	0.9751	0.9997	0.9998	0.9998	0.9995	0.9998	0.9997	0.9998	0.9998
Object rate	62.1263	15.5316	6.9029	3.7194	2.4851	1.6531	1.0143	0.9104	0.5951	0.4133
Accuracy	0.9620	0.9945	0.9999	1.0000	1.0000	0.9999	1.0000	0.9999	1.0000	1.0000
Sensitivity	0.9490	0.9961	0.9998	0.9998	0.9998	0.9994	0.9998	0.9997	0.9998	0.9998
Specificity	0.9633	0.9943	1.0000	1.0000	1.0000	0.9999	1.0000	1.0000	1.0000	1.0000
Jaccard index	0.6771	0.9515	0.9995	0.9996	0.9996	0.9989	0.9997	0.9995	0.9996	0.9997
B large										
Training time (epochs)	34	67	63	184	28	59	143	35	110	199
ERF rate before training	0.0231	0.0077	0.0087	0.0023	0.0048	0.0030	0.0049	0.0005	0.0019	0.0011
ERF rate	0.0021	0.0016	0.0006	0.0002	0.0002	0.0002	0.0001	0.0001	0.0001	0.0001
Dice score	0.8703	0.9454	0.9933	1.0000	1.0000	1.0000	1.0000	1.0000	1.0000	1.0000
Object rate	85.1332	21.2833	9.4592	5.0968	3.4053	2.2653	1.3900	1.2475	0.8155	0.5663
Accuracy	0.9368	0.9756	0.9971	1.0000	1.0000	1.0000	1.0000	1.0000	1.0000	1.0000
Sensitivity	0.7825	0.9161	0.9933	1.0000	0.9999	1.0000	1.0000	1.0000	1.0000	1.0000
Specificity	0.9943	0.9937	0.9982	1.0000	1.0000	1.0000	1.0000	1.0000	1.0000	1.0000
Jaccard index	0.7706	0.8974	0.9868	1.0000	0.9999	0.9999	1.0000	0.9999	1.0000	1.0000
B large contour										
Training time (epochs)	27	55	59	135	32	180	198	30	200	35
ERF rate before training	0.0119	0.0088	0.0064	0.0040	0.0049	0.0019	0.0040	0.0006	0.0016	0.0019
ERF rate	0.6198	0.2014	0.6863	0.0011	0.0062	0.0013	0.0027	0.0004	0.0008	0.0008
Dice score	0.6593	0.8793	0.9815	0.9998	0.9996	0.9998	0.9998	0.9994	0.9998	0.9993
Object rate	89.1812	22.2953	9.9090	5.3392	3.5672	2.3730	1.4561	1.3068	0.8543	0.5932
Accuracy	0.8838	0.9513	0.9919	0.9999	0.9998	0.9999	0.9999	0.9998	0.9999	0.9997
Sensitivity	0.9156	0.9584	0.9922	0.9998	0.9996	0.9998	0.9998	0.9995	0.9998	0.9989
Specificity	0.8795	0.9497	0.9918	0.9999	0.9999	0.9999	1.0000	0.9998	0.9999	0.9999
Jaccard index	0.4939	0.7860	0.9637	0.9996	0.9991	0.9996	0.9996	0.9989	0.9996	0.9986

### 4.1 Role of Contrast

**Fig. 5:**
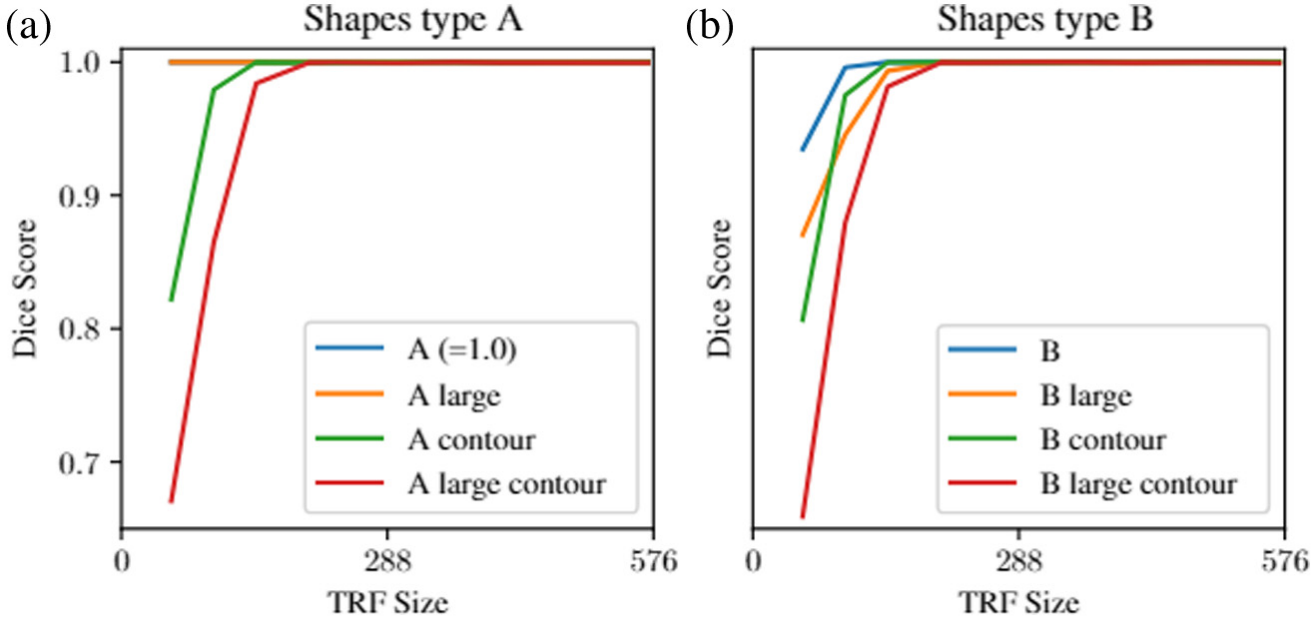
Performance of the (a) shape dataset A and (b) shape dataset B for the regular U-Net

Figure 5 illustrates the relationship between the TRF size and the DSC for all synthetic shape datasets, encompassing both types A and B for the U-Net model. For all datasets that can be segmented solely based on contrast (A, A large), the model attains perfect performance even at the smallest TRF size [Fig. 5(a)]. For datasets that present an added layer of complexity by either representing only contours of RoI in input images (A contour, A large contour) or by excluding the square from the mask (type B), a larger TRF is required to reach peak performance [Figs. 5(a) and 5(b)]. These datasets with an added complexity in segmentation show a model performance trend where DSC starts at a lower point for a small TRF and requires a larger TRF to reach peak performance, unlike the consistent perfect performance in contrast-based datasets.

**Fig. 6:**
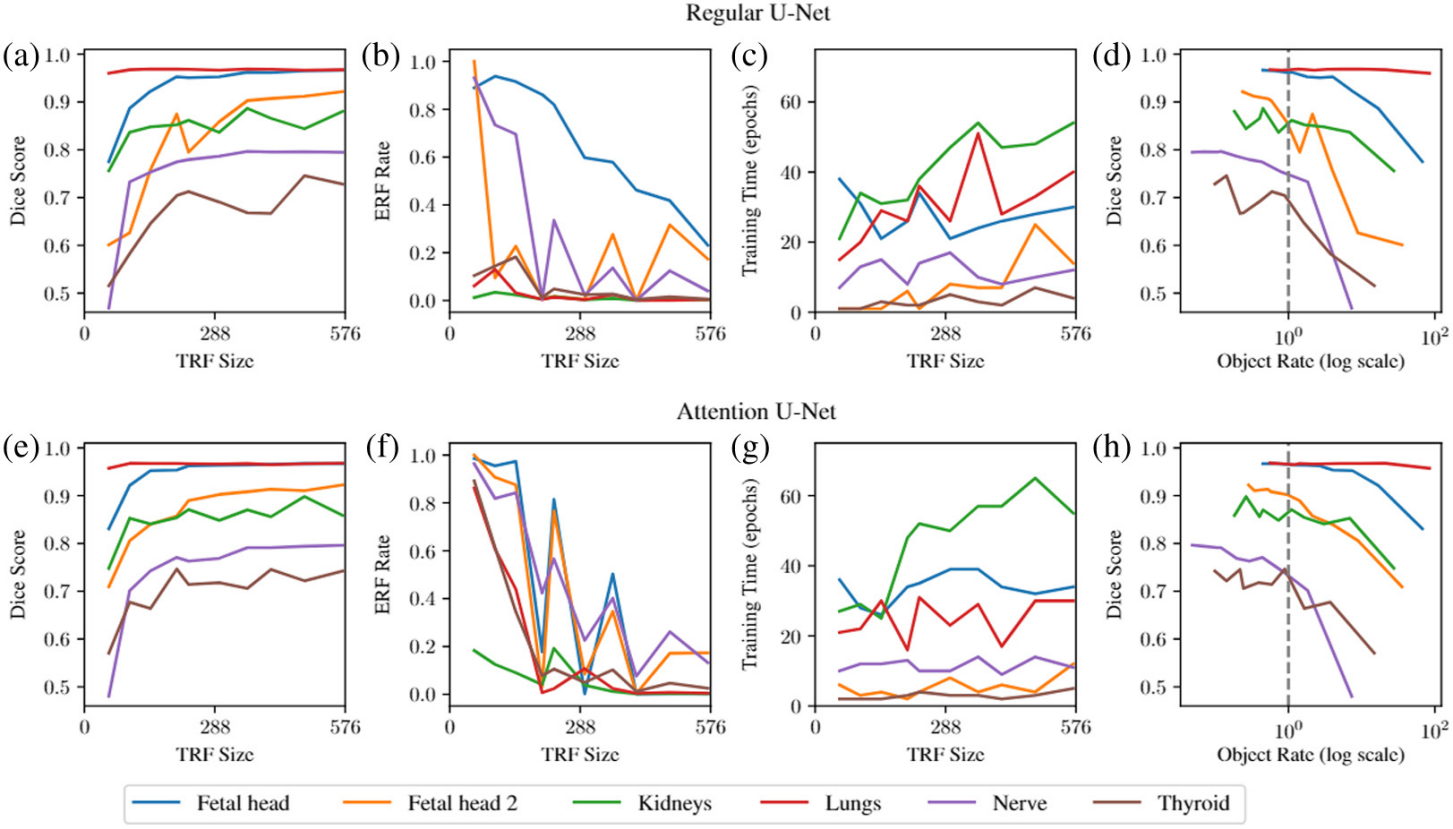
Various plots for the analyses of the medical datasets for U-Net (a) DSC versus TRF size, (b) ERF rate versus TRF size, (c) training time (epochs) versus TRF size, (d) Dice score versus object rate, and for attention U-Net, (e) DSC versus TRF size, (f) ERF rate versus TRF size, (g) training time (epochs) versus TRF size, and (h) Dice score versus object rate.

**Fig. 7:**
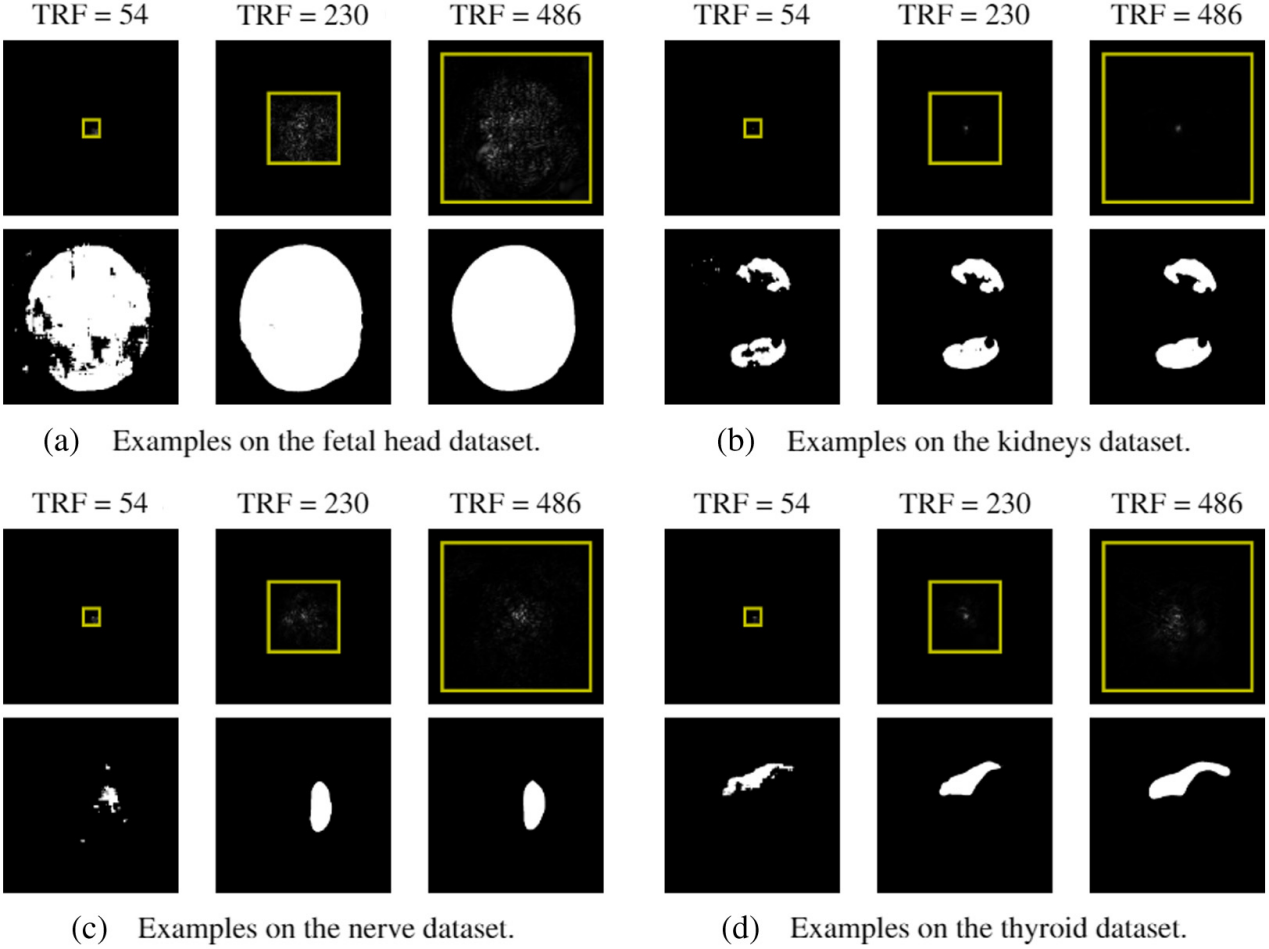
Examples of the TRF (yellow square), ERF (pixels within the TRF) in the top row in each subfigure and the predicted segmentation for various TRF sizes in the bottom row, on the samples from the fetal head (a), kidneys (b), nerve (c), and thyroid (d) datasets, as presented in Fig. 3.

**Fig. 8:**
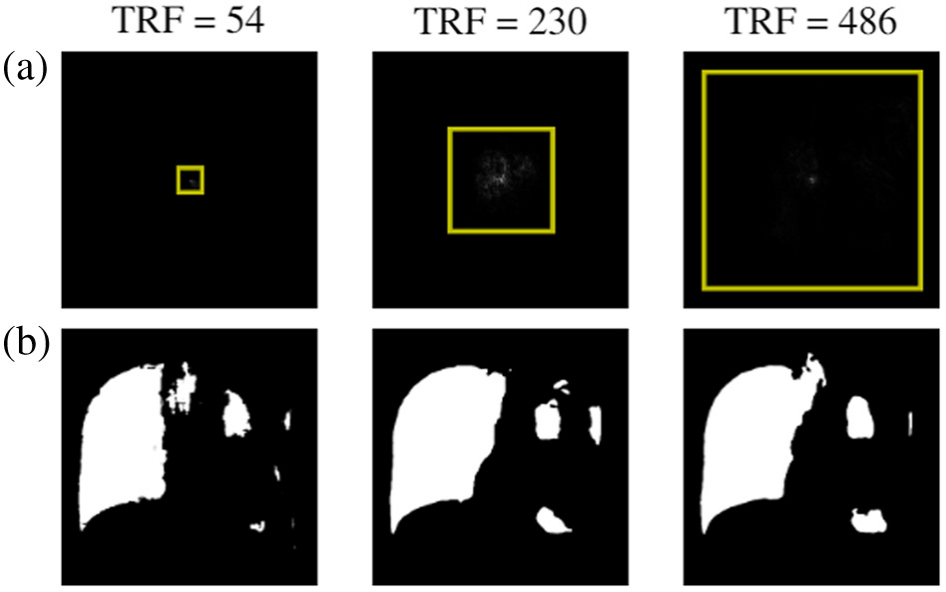
Examples of the TRF (yellow square), ERF (pixels within the TRF) in panel (a) and the predicted segmentation for various TRF sizes in panel (b), on the sample from the lungs dataset from Fig. 3.

The same pattern is present in the medical datasets: all datasets that have a low-contrast RoI show a trend of increasing DSC as the TRF size grows, whereas the high-contrast lung dataset attains peak performance starting at the lowest TRF [Figs. 6(a) and 6(e)]. The segmentation output for the datasets of fetal head, kidneys, nerve, and thyroid for different TRFs is shown in Figs. 7(a)–7(d), respectively, and the combined results for the U-net model are shown in Table 2. Figure 8 shows the results for the lung dataset for different TRFs. As, in the lung dataset, the RoI can be identified visually using the contrast, and the DSC attains close to peak value even for a very small TRF. It is clear that the predicted segmentation improves significantly with increasing TRF for all datasets except the lung dataset.

This pattern is further highlighted in Table 2, where all low-contrast datasets consistently show a trend of increasing DSC with TRF, and all high-contrast datasets do not show the same trend.

### 4.2 Optimal TRF Size

In Figs. 6(b) and 6(f), a trend is visible, which shows that the ERF rate shrinks with the enlargement of the TRF size for the U-Net and the attention U-Net respectively. This suggests that as the TRF size increases, a smaller proportion of pixels actually contribute to the predicted segmentation. Moreover, as the TRF size increases, the training time (epochs) tends to increase, as displayed in Figs. 6(c) and 6(g) for the U-Net and the attention U-Net respectively. This finding implies that an excessively large TRF size may lead to unnecessary computations in U-Net and the attention U-Net architecture, potentially explaining the observed increase in training time (epochs) with the expansion of the TRF size.

In this context, the object rate, plotted against the DSC in Fig. 6(d) (U-Net) and Fig. 6(h) (attention U-Net), also seems to play a role. When the object rate, i.e., the size of the RoI relative to the TRF, increases, the DSC degrades. This is corroborated by the two rightmost columns in Table 6, where for most low-contrast datasets where the TRF size plays a major role, the optimal TRF size, i.e., the TRF size at which the DSC saturates is usually only slightly smaller than the size of the RoI.

**Table 6:** Summary of the insights from the results. Values with no* mean that the RoI can be identified visually, but not all regions that have this contrast are included in the mask.

Dataset	Dataset type	RoI can be identified visually only by contrast	Pattern of increasing DSC with TRF	Pattern retained with attention U-Net, but higher absolute score	Average dimension of RoI	DSC saturates between TRF sizes
Nerve	Clinical	No	Yes	Yes	159	298 to 360
B contour	Synthetic	No	Yes	N/A	168	100 to 146
A contour	Synthetic	No	Yes	N/A	169	100 to 146
Thyroid	Clinical	No	Yes	Yes	187	146 to 204
B large contour	Synthetic	No	Yes	N/A	237	146 to 204
A large contour	Synthetic	No	Yes	N/A	242	146 to 204
Fetal head 2	Clinical	No	Yes	Yes	255	146 to 204
Fetal head	Clinical	No	Yes	Yes	260	146 to 204
Kidneys	Clinical	No*	Yes	Yes	101	298 to 360
B	Synthetic	No*	Yes	N/A	168	54 to 100
B large	Synthetic	No*	Yes	N/A	238	100 to 146
Lungs	Clinical	Yes	No	N/A	329	0 to 54
A	Synthetic	Yes	No	N/A	168	0 to 54
A large	Synthetic	Yes	No	N/A	244	0 to 54

Despite the overall trend of increasing DSC with expanding TRF size, we observe slight drops. This can be interpreted in light of the concept of variability in neural networks, as discussed in Ref. 29. Variability, as they defined it, represents the richness of landscape patterns in the data space with respect to well-scaled random weights. As the TRF size increases, the model starts to incorporate more global context into its predictions. Although this can be beneficial for capturing larger-scale structures in the image, it may also introduce more noise into the model’s predictions, especially if the larger TRF includes irrelevant or distracting features. This could result in slight decreases in the DSC.

### 4.3 Attention Mechanism and TRF Size

**Fig. 9:**
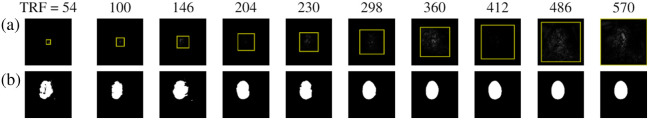
Examples of the TRF (yellow square), ERF (pixels within the TRF) in panel (a) and the predicted segmentation for various TRF sizes in panel (b), on the sample from the fetal head 2 dataset from Fig. 3.

In Figs. 6(a) and 6(e), the TRF size is plotted against the DSC for the U-Net and attention U-Net, respectively, for all the medical datasets. In both instances, the trend of an increasing DSC as the TRF grows is present. Figure 9 shows the segmentation for the fetal head 2 dataset for all the TRFs and the corresponding TRF and ERF. As the TRF size increases, the segmentation accuracy increases, and the overall trend can be seen in Table 2. The same trend is also visible for the attention U-Net model, the results of which are shown in Table 3. However, all absolute scores are higher in the case of the attention U-Net. Table 6 column 4 shows the summary of results for all the datasets if they follow the pattern of DSC using the attention U-Net model. All the medical imaging datasets except the lung dataset follow the pattern for both U-Net and attention U-Net models, with higher absolute scores for the attention counterpart. Hence, it can be said that the attention mechanism will consistently improve the performance, regardless of TRF size. Even with the attention mechanism, TRF plays an important role, and a larger TRF might further improve the performance of attention U-Net models.

### 4.4 Designing Efficient Architectures

In this work, we performed the experiments for different TRFs having the same number of total parameters for different datasets, as can be seen in Table 1. Detailed results of the performance of the U-Net model for the different metrics on all medical datasets can be found in Table 2. The results of the attention U-Net on the medical datasets and the U-Net on the synthetic datasets of types A and B can be found in Tables 3–5, respectively. These results show that even for the same number of parameters, there is a very high effect on the performance of the network if the TRF is changed. The inclusion of TRF size as a parameter for models can lead to a more fair comparison of their performance. It will also help in designing efficient architectures, ones with optimal trade-offs between performance and number of parameters.

Based on these results, we have developed some thumb rules and have incorporated them in a tool that recommends appropriate TRF size based on contrast and object size. The following are the thumb rules that the tool encompasses:

• Images that are contrast segmentable should have a TRF that need not be more than the object size.
• Images that are not contrast segmentable should have a TRF more than the object size.

## 5 Conclusion

This work highlights the essential role of the TRF size in semantic segmentation tasks with U-Net and attention U-Net architectures across datasets of various modalities. We discovered that an optimal TRF size, the one that balances the capturing of global context and computational efficiency, can significantly enhance model performance. This implies that an excessively large TRF size may lead to unnecessary computational costs without corresponding improvements in performance. In addition, our results emphasize the added value of the attention mechanism in boosting segmentation accuracy, irrespective of the TRF size.

Our findings suggest that the datasets where RoI can be visually identified by contrast comparison alone typically attain peak performance with even small TRF. Conversely, this is not the case when additional complexities are present, such as contrast not being the only criteria for identifying RoI or contours demarcating RoI. This implies that the model’s performance also depends on factors such as the complexity of the task and the size of the RoI relative to the TRF size.

Furthermore, our study indicates that the DSC tends to plateau at a certain TRF size depending on the dataset. This suggests that there exists an optimal TRF size for each dataset, beyond which further expansion of the TRF size does not significantly improve the DSC. These findings can have practical implications for the design of segmentation models, suggesting that increasing TRF size may not always be necessary or beneficial.

These insights provide a valuable reference for designing and optimizing U-Net-based architectures for various tasks and datasets in medical imaging. Although our study focused on the U-Net and attention U-Net architectures, there are many other architectures used for semantic segmentation tasks, such as SegNet,^30^ PSPNet,^31^ and DeepLab.^32^ Future research could investigate the impact of the TRF size on the performance of these architectures.

## 6 Appendix A: Convolution

In a 2D convolution layer, a filter or kernel is applied to a 2D image, performing a dot product at each position.^33^ The kernel size (k) impacts the detail level captured, whereas the stride (s) affects the kernel shift amount. Padding (p), set to “same” in this study, ensures that the output feature map matches the input image dimensions, permitting edge-based convolution operations.

If the padding is set to same, the number of arrays that must be padded on every side of the $T^{\left(d-1\right)}$ tensor to simulate a convolution while maintaining the previous layer’s dimensions can be calculated. For a h×w layer, the padding values along the y and x axes are computed as follows:

$$p_{y}=\lfloor \frac{(h-1)\cdot s+k-h}{2}\rfloor ,$$ (9)

$$p_{x}=\lfloor \frac{(w-1)\cdot s+k-w}{2}\rfloor .$$ (10)

Therefore, along the first and second axes of the four-dimensional tensor $T^{\left(d-1\right)}$, the tensor is padded with $p_{y}$ and $p_{x}$, two-dimensional tensors that contain the same values as the edges along the first and second axes of $T^{\left(d-1\right)}$. Let $P^{\left(d-1\right)}$ denote this padded tensor. For each position (i,j), the top-left and bottom-right pixels from the previous layer’s TRF can be fetched from $P^{\left(d-1\right)}$ at the indices (i·s,j·s) and (i·s+k−1,j·s+k−1), respectively. Thus, the TRF at position (i,j) for a convolutional layer at depth d can be denoted as

$$T_{i,j}^{\left(d\right)}=[\begin{array}{ll} P_{i\cdot s,j\cdot s,0,0}^{\left(d-1\right)} & P_{i\cdot s,j\cdot s,0,1}^{\left(d-1\right)}\\ P_{i\cdot s+k-1,j\cdot s+k-1,1,0}^{\left(d-1\right)} & P_{i\cdot s+k-1,j\cdot s+k-1,1,1}^{\left(d-1\right)} \end{array}].$$ (11)

## 7 Appendix B: Max Pooling

2D max pooling is a feature map reduction method where a rectangular kernel selects maximum values within regions, creating a smaller feature map.^33^ The kernel size (k) defines the sliding window size over the input, and the stride (s)—in our study equal to k to simplify the computation—controls the window’s movement.

For a given position (i,j), the topmost and leftmost pixels from the previous layer’s TRF can be accessed from the $T^{\left(d-1\right)}$ tensor at the index of (i·k,j·k), whereas the bottom-most and rightmost pixels can be accessed at the index of (i·k+k−1,j·k+k−1). As such, the TRF at position (i,j) for a max pooling layer at depth d can be expressed as follows:

$$T_{i,j}^{\left(d\right)}=[\begin{array}{cc} T_{i\cdot k,j\cdot k,0,0}^{\left(d-1\right)} & T_{i\cdot k,j\cdot k,0,1}^{\left(d-1\right)}\\ T_{i\cdot k+k-1,j\cdot k+k-1,1,0}^{\left(d-1\right)} & T_{i\cdot k+k-1,j\cdot k+k-1,1,1}^{\left(d-1\right)} \end{array}].$$ (12)

## 8 Appendix C: Upsampling

Upsampling is a technique used to increase the spatial resolution of feature maps. In particular, it is implemented through transposed convolution or deconvolution, which is the reverse operation of convolution. During the transposed convolution operation, a kernel of size k is applied to the input feature map to generate an output feature map with a higher spatial resolution. The stride s determines the amount of shift in the output feature map for each kernel application.^33^ When the stride is set to k, the size of the output feature map is equal to the size of the input feature map multiplied by the stride.

However, when the stride s is different from the kernel size k, there may be overlaps in the values of the output feature map. Therefore, an iterative method is required to identify the corners of the TRF for each pixel in the output feature map. Specifically, Algorithm 1 is applied to each pixel (i,j) in the input map, computing the range in which the pixel is copied to the output feature map by multiplying the top and left indices with the stride and the bottom and right indices with the stride and then adding the kernel size. The algorithm then iterates over the pixels (m,n) in the output feature map within this range. If there is no overlap, the indices from the previous layer at (i,j) are simply copied. Otherwise, for the top and left of the TRF, the algorithm takes the minimum of the current index and a potentially overlapping index, whereas for the bottom and right TRF, it takes the maximum.

**Algorithm 1:** TRF at layer d and pixel (i,j) after upsampling

**for**m←i·s to i·s+k **do**
**for** n←j·s to j·s+k **do**
**if** $T_{m,n}^{\left(d\right)}$ **is** None **then** ▹ If no overlap (yet)
$T_{m,n}^{\left(d\right)}\leftarrow T_{i,j}^{\left(d-1\right)}$ ▹ Copy from previous layer
**continue** ▹ Go to next pixel
**end if**
**if** $T_{i,j,0,0}^{\left(d-1\right)}\leq T_{m,n,0,0}^{\left(d\right)}$ **then** ▹ If there is overlap:
▹ Get smallest value
$T_{m,n,0,0}^{\left(d\right)}\leftarrow T_{i,j,0,0}^{\left(d-1\right)}$ ▹ Update top
**end if**
**if** $T_{i,j,0,1}^{\left(d-1\right)}\leq T_{m,n,0,1}^{\left(d\right)}$ **then** ▹ Get smallest value
$T_{m,n,0,1}^{\left(d\right)}\leftarrow T_{i,j,0,1}^{\left(d-1\right)}$ ▹ Update left
**end if**
**if** $T_{i,j,1,0}^{\left(d-1\right)}\geq T_{m,n,1,0}^{\left(d\right)}$ **then** ▹ Get largest value
$T_{m,n,1,0}^{\left(d\right)}\leftarrow T_{i,j,1,0}^{\left(d-1\right)}$ ▹ Update bottom
**end if**
**if** $T_{i,j,1,1}^{\left(d-1\right)}\geq T_{m,n,1,1}^{\left(d\right)}$ **then** ▹ Get largest value
$T_{m,n,1,1}^{\left(d\right)}\leftarrow T_{i,j,1,1}^{\left(d-1\right)}$ ▹ Update right
**end if**
**end for**
**end for**

## 9 Appendix D: Concatenations

Within the U-Net architecture, skip connections from layer d−c are integrated into the decoder blocks by concatenating them with the output of the upsampling layer d−1.^34^ To achieve this, the TRF of the tensors being concatenated, denoted as $T_{i,j}^{\left(d-c\right)}$ and $T_{i,j}^{\left(d-1\right)}$, must first be determined. The TRF of each pixel after concatenation, denoted as $T_{i,j}^{\left(d\right)}$, is obtained by selecting the lowest indices for the left and top of both TRFs and the highest indices for the right and bottom of both TRFs. This approach ensures that the largest possible TRF is obtained:

$$T_{i,j,0,0}^{\left(d\right)}=\min(\{T_{i,j,0,0}^{\left(d-1\right)},T_{i,j,0,0}^{\left(d-c\right)}\}),$$

$$T_{i,j,0,1}^{\left(d\right)}=\min(\{T_{i,j,0,1}^{\left(d-1\right)},T_{i,j,0,1}^{\left(d-c\right)}\}),$$

$$T_{i,j,1,0}^{\left(d\right)}=\max(\{T_{i,j,1,0}^{\left(d-1\right)},T_{i,j,1,0}^{\left(d-c\right)}\}),$$

$$T_{i,j,1,1}^{\left(d\right)}=\max(\{T_{i,j,1,1}^{\left(d-1\right)},T_{i,j,1,1}^{\left(d-c\right)}\}).$$

## 10 Appendix E: Activation Functions

Although nonlinear activation functions such as ReLU and sigmoid do affect the ERF by potentially reducing its size when certain parameters are set to zero,^35^ they have no effect on the TRF as these functions act element-wise on the previous layer. Therefore, in a layer d with an activation function, it can be concluded that $T_{i,j}^{\left(d\right)}=T_{i,j}^{\left(d-1\right)}$.

## 11 Appendix F: Attention Gates

Attention gates, a key component of the attention U-Net architecture (illustrated in Fig. 1), receive input features from a layer denoted as $x^{\prime }$ and a gating signal from a layer g.^5^ The inputs are then subjected to 1×1 convolutions, followed by element-wise addition. At this point, the TRF is equivalent to the maximum range of the TRF of either input as the TRF is not modified by the 1×1 convolution. Next, a ReLU and sigmoid function are applied, which leave the TRF unchanged, as described in Appendix E. Finally, element-wise multiplication is performed on the output, which results in the TRF being equivalent to the maximum range of the TRF of either input. As a result, the TRF sizes of a U-Net and attention U-Net with the same depth and convolution kernel sizes are equivalent.

Therefore, similar to concatenations, the TRF of an attention gate a is the maximal range of the TRF from its input features of layer $x^{\prime }$ and the gating signal of layer g:

$$T_{i,j,0,0}^{\left(a\right)}=\min(\{T_{i,j,0,0}^{\left(x^{\prime }\right)},T_{i,j,0,0}^{\left(g\right)}\}),$$

$$T_{i,j,0,1}^{\left(a\right)}=\min(\{T_{i,j,0,1}^{\left(x^{\prime }\right)},T_{i,j,0,1}^{\left(g\right)}\}),$$

$$T_{i,j,1,0}^{\left(a\right)}=\max(\{T_{i,j,1,0}^{\left(x^{\prime }\right)},T_{i,j,1,0}^{\left(g\right)}\}),$$

$$T_{i,j,1,1}^{\left(a\right)}=\max(\{T_{i,j,1,1}^{\left(x^{\prime }\right)},T_{i,j,1,1}^{\left(g\right)}\}).$$

## Data Availability

The complete source code utilized in this work can be accessed via our GitHub repository at https://github.com/vinloo/u-net-receptive-field-study. In addition to this, we have developed an open-source tool designed to calculate and suggest an appropriate TRF size based on a specified U-Net configuration and dataset. This tool is intended to aid researchers and practitioners in the field and is included in the repository.

## References

[1] Litjens G. et al., “A survey on deep learning in medical image analysis,” Med. Image Anal. 42, 60–88 (2017). 10.1016/j.media.2017.07.005 28778026

[2] Hesamian M. H. et al., “Deep learning techniques for medical image segmentation: achievements and challenges,” J. Digit. Imaging 32, 582–596 (2019). 10.1007/s10278-019-00227-x 31144149 PMC6646484

[3] Ronneberger O. Fischer P. Brox T., “U-Net: convolutional networks for biomedical image segmentation,” Lect. Notes Comput. Sci. 9351, 234–241 (2015). 10.1007/978-3-319-24574-4_28

[4] Williams C. et al., “A unified framework for U-Net design and analysis,” Adv. Neural Inf. Process. Syst. 36, 27745–27782 (2023).

[5] Oktay O. et al., “Attention U-Net: learning where to look for the pancreas,” arXiv:1804.03999 (2018).

[6] Luo W. et al., “Understanding the effective receptive field in deep convolutional neural networks,” Adv. Neural Inf. Process. Syst. 29 (2017).

[7] Araujo A. Norris W. Sim J., “Computing receptive fields of convolutional neural networks,” Distill (2019). 10.23915/distill.00021

[8] Behboodi B. et al., “Receptive field size as a key design parameter for ultrasound image segmentation with U-Net,” in 2020 42nd Ann. Int. Conf. IEEE Eng. Med. Biol. Soc. (EMBC), pp. 2117–2120 (2020). 10.1109/EMBC44109.2020.9175846 33018424

[9] Sytwu K. Groschner C. Scott M. C., “Understanding the influence of receptive field and network complexity in neural network-guided TEM image analysis,” Microscopy Microanalysis 28, 1896–1904 (2022). 10.1017/S1431927622012466 36097787

[10] Yu F. Koltun V., “Multi-scale context aggregation by dilated convolutions,” (2016).

[11] Sarıgül M. Ozyildirim B. Avci M., “Differential convolutional neural network,” Neural Networks 116, 279–287 (2019). 10.1016/j.neunet.2019.04.025 31125914

[12] Paszke A. et al., “Pytorch: an imperative style, high-performance deep learning library,” Adv. Neural Inf. Process. Sys. 32, 8026–8037 (2019).

[13] Zhang Z., “Improved Adam optimizer for deep neural networks,” in 2018 IEEE/ACM 26th Int. Symp. Quality of Service (IWQoS), pp. 1–2, IEEE (2018).

[14] Prechelt L., “Early stopping-but when?” in Neural Networks: Tricks of the Trade, pp. 55–69, Springer (2002).

[15] van den Heuvel T. L. A. et al., “Automated measurement of fetal head circumference using 2D ultrasound images,” PloS one 13(8), e0200412 (2018). 10.1371/journal.pone.0200412 30138319 PMC6107118

[16] Lu Y. et al., “JNU-IFM,” Data in Brief 41, 107904 (2022). 35198683 10.1016/j.dib.2022.107904 PMC8842023

[17] Lu Y. et al., “The JNU-IFM dataset for segmenting pubic symphysis-fetal head,” Data Brief 41, 107904 (2022). 10.1016/j.dib.2022.107904 35198683 PMC8842023

[18] Daniel A. J. et al., “T2-weighted kidney MRI segmentation,” Zenodo (2021).

[19] Daniel A. J. et al., “Automated renal segmentation in healthy and chronic kidney disease subjects using a convolutional neural network,” Magn. Reson. Med. 86, 1125–1136 (2021). 10.1002/mrm.28768 33755256

[20] Danilov V., “Chest x-ray dataset for lung segmentation,” Mendeley Data 10 (2022).

[21] Kassamali R. H. Jafarieh S., “Passion and hard work produces high quality research in UK: response to focus on China: should clinicians engage in research? and lessons from other countries,” Quant. Imaging Med. Surg. 4(6), 502–503 (2014). 10.3978/j.issn.2223-4292.2014.11.21 25525585 PMC4256237

[22] Wunderling T. et al., “Comparison of thyroid segmentation techniques for 3D ultrasound,” Proc. SPIE 10133, 1013317 (2017). 10.1117/12.2254234

[23] Anna Montoya W. C., “Ultrasound nerve segmentation,” https://kaggle.com/competitions/ultrasound-nerve-segmentation (2016).

[24] Setiawan A. W., “Image segmentation metrics in skin lesion: accuracy, sensitivity, specificity, dice coefficient, Jaccard index, and Matthews correlation coefficient,” in 2020 Int. Conf. Comput. Eng. Network, Intell. Multimedia (CENIM), pp. 97–102 (2020).

[25] Müller D. Soto-Rey I. Kramer F., “Towards a guideline for evaluation metrics in medical image segmentation,” BMC Res. Notes 15, 210 (2022). 10.1186/s13104-022-06096-y 35725483 PMC9208116

[26] Stanislaw W., “Kernel density estimation and its application,” ITM Web Conf. 23, 00037 (2018). 10.1051/itmconf/20182300037

[27] Silverman B. W., Density Estimation for Statistics and Data Analysis, pp. 47–48, Chapman & Hall, London (1986).

[28] Harpole J. K. et al., “How bandwidth selection algorithms impact exploratory data analysis using kernel density estimation,” Psychological Methods 19, 428–443 (2014). 24885339 10.1037/a0036850

[29] Yu Y. Zhang Y., “Multi-layer perceptron trainability explained via variability,” (2023).

[30] Badrinarayanan V. Kendall A. Cipolla R., “SegNeT: a deep convolutional encoder-decoder architecture for image segmentation,” IEEE Trans. Pattern Anal. Machine Intell. 39(12), 2481–2495 (2017). 10.1109/TPAMI.2016.2644615 28060704

[31] Zhao H. et al., “Pyramid scene parsing network,” in Proc. IEEE Conf. Comput. Vision Pattern Recognit., pp. 2881–2890 (2017).

[32] Chen L.-C. et al., “DeepLAB: semantic image segmentation with deep convolutional nets, atrous convolution, and fully connected CRFS,” IEEE Trans. Pattern Anal. Mach. Intell. 40(4), 834–848 (2017). 10.1109/TPAMI.2017.2699184 28463186

[33] Dumoulin V. Visin F., “A guide to convolution arithmetic for deep learning,” arXiv:1603.07285 (2018).

[34] Wu J. et al., “Skip connection U-Net for white matter hyperintensities segmentation from MRI,” IEEE Access 7, 155194–155202 (2019). 10.1109/ACCESS.2019.2948476

[35] Kuo C.-C. J., “Understanding convolutional neural networks with a mathematical model,” J. Vis. Commun. Image Represent. 41, 406–413 (2016). 10.1016/j.jvcir.2016.11.003

